# On the Healing of Wounds from Burns and Scalds, by the Scabbing Process

**Published:** 1825-09

**Authors:** F. Bush

**Affiliations:** Surgeon.


ART. III.—*On the Healing of Wounds from Burns and Scalds, by the Scabbing Process.* By F. BUSH, Esq. Surgeon.

THE cicatrisation of surfaces extensively denuded of their natural covering, such as frequently occur from burns and scalds, is generally a subject of some embarrassment, when treated in the ordinary method, and, from the tardiness of the operation, is productive of much pain and inconvenience to the subjects of them. This is more particularly the case when such injuries are inflicted on the trunk; the healing of such sores on the extremities is effected with less difficulty, as they can be placed under the influence of pressure, which cannot be so well employed to aid cures of this kind on the back, abdomen, chest, or neck. Indeed, I have seen the most severe and lengthened distress arise from such injuries; the rapid growth of soft flabby granulations proving not only difficult to restrain, but retarding the skinning process, the sore pouring out large quantities of pus, and affecting the general health, so as to induce hectic fever, and even fatal consequences.

As the object of my paper is to call the attention to that stage of the injury which is secondary, I shall only advert to recent injuries of this sort, by offering my humble tribute of approval of the plan laid down by Dr. Kentish; but as, where violent heat has been applied, notwithstanding the use of the most approved remedies, sloughing of the skin will take place, I shall take up the subject just at that stage when the sore has put on a healthy granulating surface. Perhaps my plan of treatment will best admit of illustration by the recital of a few cases.

CASE I.—About seven years ago, a child of J. Burge was brought to me with a sore on the back, as large as a man's hand: it was situated just below the right scapula; the surface was uneven, and the granulations morbidly soft, throwing out much pus, and bleeding on the slightest touch. I learnt that it had arisen from a burn, and had been very much in its then-present state for two years, notwithstanding it had been under the care of a most respectable surgeon for some time, and had been subsequently dressed by various healing ointments, used empirically on such occasions. I determined that nothing of an unctuous character should be used, but that the edges of the sore should be touched daily with the liq. plumbi, applied by a camel's-hair brush, and that the whole surface should be sprinkled over with flour or chalk, so as to form an artificial scab. Some portion of the scab was daily broken down, the matter discharged, and more flour used to promote the formation of a new scab. During the cure the child wore a loose dress, like a petticoat tied round the neck, and every thing, but the application before stated, kept as much as possible from coming in contact with the sore: by these means, complete cicatrisation had taken place in a month.

CASE II.—Miss Hooper, a young lady about twelve years of age, was dreadfully burnt on the neck and back, from her clothes taking fire: the injury was so severe that, for the week after the accident, her death was expected; but the constitution was supported by stimulants and opium, and at length a slough, of a most frightful extent, was thrown off. For some time, calamine cerate, and an ointment prepared from oxyde of lead, were used to dress the wound; but the discharge became so considerable, the granulation so redundant, and the case attended with so much constitutional disorder, that we again had reason to fear a fatal termination. Under these unfavourable circumstances, I had recourse to the use of the liquor plumbi, with which the edges of the sores were daily smeared, and wheaten flour sprinkled on the granulations, so as to encourage the formation of a scab. This plan was persevered in for a few weeks, the scab being occasionally broken down, and a new one formed in the same manner; the health soon improved; and the sore healed in half the time that I have seen required to accomplish the same object under the ordinary method of treatment.

CASE III.—J. Ashby, a boy about fourteen years of age, had his side severely burnt, from a squib taking fire in his pocket: the heat was so intense, and so long applied, that there was considerable sloughing. After that had taken place, I had recourse to the liquor plumbi and flour; and the progress of the cure was so rapid, that both myself and patient's friends were perfectly satisfied with the superior efficacy of the plan of treatment.

I would add one remark on the treatment of scalds of the extremities, such as I have often witnessed on the feet and ankles of cooks and housemaids. When blistering has taken place, much relief is found from puncturing the bladders, and binding the limb evenly, and rather firmly, with strips of linen spread with emplastrum plumbi, on the plan recommended by Baynton for the cure of ulcerated legs. I have observed that the dressings do not require to be removed daily,—once in two days has answered with me; and, if the parts have become painful, cloths wet with cold water have been from time to time employed with advantage, wrapped round the limb.

*Frome; 13th July,* 1825.

